# Proteomic Analysis of Red Ginseng on Prolonging the Life Span of Male *Drosophila melanogaster*

**DOI:** 10.3389/fphar.2021.618123

**Published:** 2021-06-11

**Authors:** Wei Hou, Jin Pei

**Affiliations:** ^1^ School of Pharmaceutical Sciences, Jilin University, Changchun, China; ^2^ Institute of Special Animal and Plant Sciences, Chinese Academy of Agricultural Sciences, Changchun, China

**Keywords:** red ginseng, anti-Aging, isobaric tag for relative and absolute quantitation, proteome, drosophila

## Abstract

Ginseng (*Panax ginseng* C. A. Mey.) is a traditional medicine that has been utilized for over 2000 years in Asia and shows varied pharmacological effects. Red ginseng (RG) is steamed and dried ginseng root and is considered to be more effective. Heating inactivates its catabolic enzymes and increases the activities of RG, which can improve the immune system, alleviate fatigue, and has anti-inflammatory effects and antioxidant activity. In addition, RG has a good anti-aging effect, but its mechanism is unclear. Senescence, a side-effect of normal developmental and metabolic processes, is a gradual decline in physiological integrity and function of the body. Senescence is usually associated with a variety of diseases, including neurodegenerative diseases and diabetes. Research on anti-aging and the prolongation of life span has always been a focus topic. In this study, we investigated the molecular mechanism of RG that results in prolonged the life span for male *Drosophila melanogaster.* Isobaric tag for relative and absolute quantitation (iTRAQ) was used to identify protein changes in an old male *D. melanogaster* treated with RG. The differential proteins were verified by qRT-PCR and western blotting. The results showed that 12.5 mg/ml RG prolonged its life span significantly. iTRAQ results showed that, compared to the control group, 32 upregulated proteins and 62 downregulated proteins displayed significantly differential expression in the RG group. In this study, we explored the pathways that RG may participate in that extend the life span of *D. melanogaster*, and the results showed that the PI3K/AKT/FoxO pathway was involved. In addition, 4E-BP increased and participated in the regulation of life span.

## Introduction

Senescence is a gradual decline in the physiological integrity and function of the body, including molecules, cells, tissue structure, and function, as well as homeostasis (He and Jasper, 2014). Senescence is usually associated with various diseases, such as neurodegenerative diseases, diabetes, cardiovascular diseases, and cerebrovascular diseases. Research on anti-aging and the prolongation of life span has always been a point of focus.

Ginseng (*Panax ginseng* C. A. Mey.) is a traditional medicine that has been utilized for over 2000 years in Asia and has varied pharmacological effects (Wisniewski et al., 2009; Wu et al., 2017). The ancient Chinese Meteria Medica *ShenNong BenCao Jing* recorded that ginseng could be taken for a long time and prolong life. Modern pharmacological studies have shown that ginseng extract enhances the activity of superoxide dismutase in aged rats (Ramesh et al., 2012). In addition, ginseng has been shown to be cardioprotective because of its antioxidative, anti-arrhythmic, and calcium channel-antagonistic activities (Yuan 2015). Red ginseng (RG) is steamed and dried ginseng root, which is considered more effective. Heating inactivates its catabolic enzymes and increases the activities of RG, which can improve the immune system and alleviate fatigue and has anti-inflammatory effects and antioxidant activity; it allows effective coping with the metabolic dysfunction of senescent cells and functional decline (Wu et al., 2017; Ham et al., 2019). Furthermore, RG can protect the brain and spinal cord from neurodegeneration and extend the life span of *Drosophila melanogaster* (Wisniewski et al., 2009; Kim 2013; Liu et al., 2018).

*D. melanogaster* is an excellent model for research on senescence due to its short life span and easy maintenance (Allocca et al., 2018). In our previous experiments, we fed *D. melanogaster* RG, and the results showed that RG extended the life span of *D. melanogaster* in both males and females (Liu et al., 2018; Hou et al., 2020). However, the mechanism by which RG extends the life span of male *D. melanogaster* has not been elucidated. In this study, an isobaric tag for relative and absolute quantitation (iTRAQ) was used to identify protein changes in senile male *D. melanogaster* treated with RG and is the first study to reveal the changes in signaling pathways.

## Materials and Methods

### Materials

**FIGURE 1:**
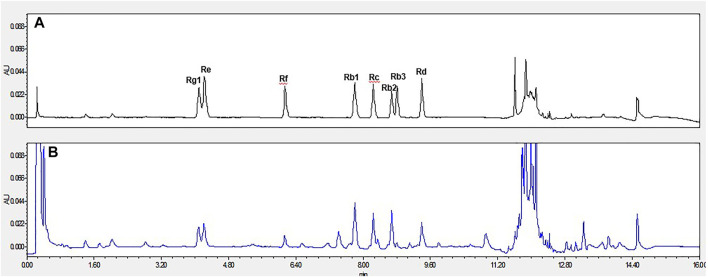
The representative UPLC chromatograms of ginsenosides. **(A)**. The reference standards of ginsenosides Rg1, Re, Rf, Rb1, Rc, Rb2, Rb3, Rd. **(B)**. Red ginseng sample.; Abbreviation: UPLC, Ultra Performance Liquid Chromatography.

Red ginseng (6 years) was purchased from Changchun City (Jilin Province, China) and pulverized into a powder. The panaxoside content was determined by high-performance liquid chromatography method (Zhang et al., 2018), and the contents were (all in mg/g) Re 1.470, Rg1 1.836, Rf 1.01, Rb1 5.21, Rc 4.447, Rb2 3.211, Rb3 0.317, and Rd 4.453 (Figure 1).

### Red Ginseng Prolongs the Life Span of Male *D. melanogaster*

*D. melanogaster* (wild type) were a gift from Jilin Agricultural University (Changchun, China). All male *D. melanogaster* were housed in an artificial climate incubator at 25°C and 60% humidity with 12 h alternating dark and light phases. New eclosion *D. melanogaster* were separated by sex and males were subsequently divided into six groups (*n* = 60 each). *D. melanogaster* of the control group were fed a basic diet of water, agar, corn extract, sucrose, extra yeast powder, and propionic acid, and *D. melanogaster* of the RG group were fed basic diet supplemented with RG at a final concentration of 10.0, 12.5, 15.0, 17.5, or 20.0 mg/ml. The food was changed every 2–3 days. Survival was recorded at 8:00 AM for both the control and RG groups. *D. melanogaster* that died unnaturally were eliminated (accidental death), and the *D. melanogaster* that died naturally were removed from the cage. The test was repeated three times. All animals were handled in strict accordance with good animal practice according to the Animal Ethics Procedures and Guidelines of the People’s Republic of China, and the study was approved by The Animal Administration and Ethics Committee of Institute of Special Animal and Plant Sciences, Chinese Academy of Agricultural Sciences.

### Climbing Ability Test

The male *D. melanogaster* of the control and RG groups were tested at the age of 36 days. The test was repeated three times. The locomotor activity of *D. melanogaster* was measured by climbing ability. *D. melanogaster* were placed into culture tubes (Diameter, 2.5 cm; Height, 12 cm). *D. melanogaster* was tapped down to the bottom of tube and then the maximum height that the *D. melanogaster* climbed up the tube was measured over 15 s (Iliadi and Boulianne 2010; Minois et al., 2001).

### Protein Preparation

The male *D. melanogaster* of the control and RG group were anesthetized at the age of 36 days. Liquid nitrogen was added to the samples (The male *D. melanogaster* body) and ground into a fine powder. Then, the powder was dissolved in SDT buffer (4% sodium dodecyl sulfate, 0.1 M; dithiothreitol, 100 mM; Tris-HCl, pH 7.6). The protein content was tested using a BCA protein assay (Thermo Fisher Scientific, United States). Protein digestion was performed according to the filter-aided proteome preparation procedure (Wisniewski et al., 2009). The peptides were desalted on MILI-SPE Extraction disk cartridge (C18-SD), lyophilized, and 40 µL dissolution buffer was added.

### Isobaric Tag for Relative and Absolute Quantitation Labeling

Each peptide mixture (100 μg) was labeled with the iTRAQ reagent-8 plex Multiplex Kit (AB SCIEX United Kingdom. Limited, United Kingdom). The peptide mixture of control-male-1 was labeled as a 113 tag. Control-male-2 was labeled as a 114 tag, and control-male-3 was labeled as a 115 tag. RG-male-1 was labeled as a 116 tag, RG-male-2 as a 117 tag, and RG-male-3 as a 118 tag.

### LC-MS/MS Analysis

Each peptide mixture was analyzed by nano LC-MS/MS coupled to an EASY nLC (Thermo Fisher Scientific). The sample was loaded into the column (Thermo Scientific Acclaim PepMap100, 100 μm × 2 cm, nanoViper C18) by an automatic sampler and connected to an analytical column (Thermo Scientific EASY Column, 10 cm, ID75 μm, 3 μm, C18-A2) in buffer A (0.1% formic acid) and buffer B (84% acetonitrile and 0.1% formic acid) at a flow rate of 300 nL/min. LC-MS/MS analysis was performed on a Q Exactive mass spectrometer (Thermo Scientific). The mass spectrometer was detected in positive ion mode, and the precursor ions scanning range was 300–1,800 m/z. The automatic gain control (AGC) target was set to 1e6 and maximum inject time (IT) to 50 ms. Survey scans were acquired at a resolution of 70,000 at m/z 200 and the resolution for HCD spectra was set to 17,500 at m/z 200, and an isolation width of 2 m/z. Normalized collision energy was 30 eV, and the underfill ratio was defined as 0.1% (Liu et al., 2017; Yu et al., 2017). Raw mass spectrometry data were submitted to China National Genomics Data Center (CNCB-NGDC, https://bigd.big.ac.cn/omix/) BioProject accession number under PRJCA004725.

### Proteomic Analysis

The mass spectrometry data were noted and quantified using the MASCOT engine (version 2.2; Matrix Science, London, United Kingdom) and Proteome Discoverer 1.4 (Thermo Fisher Scientific). The selected database was UniProt *D. melanogaster* 42524 20180327. fasta. The following options were used to identify proteins: peptide mass tolerance = ±20 ppm; fragment mass tolerance = 0.1 Da; enzyme = trypsin; max missed cleavages = 2; fixed modification: carbamidomethyl (C), iTRAQ4/8plex (N-term), iTRAQ4/8plex (K); and variable modification: xidation (M), iTRAQ4/8plex (Y); database pattern = decoy. The selection criteria for differential proteins were set as fold-change with a comparison >1.2 or <0.83, combined with an unadjusted *p* < 0.05. The Blast2GO program (https://www.blast2go.com/) was used to annotate the functions of the differentially expressed proteins. The Kyoto Encyclopedia of Genes and Genomes (KEGG) database was used to analyze the pathway enrichment of significant proteins (Liu et al., 2017; Yu et al., 2017).

### qRT-PCR

The identified differentially expressed proteins were examined at the transcriptional level by qRT-PCR. Glyceraldehyde-3-phosphate dehydrogenase (GAPDH; Proteintech, United States) was used as an internal reference. *D. melanogaster* from the control and RG group were euthanized at the age of 36 days. The test was repeated three times. Total RNA was extracted using the Total RNA Extraction Kit (UNIQ-10 Trizol, SK1321, Shanghai, China), and cDNA was synthesized using the Thermo Fisher Scientific cDNA Synthesis kit (EP0733). The 2^−ΔΔCT^ method was used to analyze RNA expression. Primer sequences for qRT-PCR are shown in Table 1.

**TABLE 1:** Primer sequences.

<Gene	Primer sequences
GAPDH	F 5' CCT​ATG​ACG​AAA​TCA​AGG​CTA​A 3'
	R 5' GCT​GAA​GAA​GTC​GGT​GGA​GA 3'
CG9286	F 5' TGC​CCA​GGG​AAT​CTC​TCA​A 3'
	R 5' GCA​CAG​AGG​TGA​TTC​CAA​AGA​T 3'
Vir-1	F 5' CGA​CTG​GCG​ACT​CTG​ATG​AT 3'
	R 5' AAT​GGT​GGA​AGT​GGT​GTT​GG 3'
CG10472	F 5' TTC​GGT​CAA​GAA​CCT​GAA​CAT​C 3'
	R 5' TTGGGCTCGGCAATCTGT 3'
Rheb	F 5' AAA​ACT​GCT​CGA​CGT​AAT​GGG 3'
	R 5' CCC​ACG​GAC​TCG​TTC​TGT​TT 3'
Ent2	F 5' GAG​TTA​CCG​CAC​CCA​TTT​CA 3'
	R 5' AGGTCGCCGCCAAAGTT 3'
ND-MLRQ	F 5' TCA​CTT​GGA​ATC​GCA​CAT​CA 3'
	R 5' TAG​TTT​TGG​AAT​AAT​CCC​TCA​CAG 3'
Rab4	F 5' GAT​CGT​ACT​ATC​GCG​GAG​CA 3'
	R 5' GCA​TCA​TTC​AGC​CAG​TTT​GTC 3'
Path	F 5' ACA​ATC​CGC​ATC​CAA​CAA​CC 3'
	R 5' GCA​ATT​TCG​GCA​AAG​GTC​AT 3'
Muskelin	F 5' GCC​AGC​AGG​GCT​ATT​TGA​GT 3'
	R 5' TTG​CCA​GGC​TTT​ATG​TCG​C 3'
FK506-bp2	F 5' AAA​AGG​TCA​CGG​TCC​ACT​ACA​C 3'
	R 5' AAC​TGG​GCA​ACT​CCC​TCA​TC 3'
Akh	F 5' GAA​TCC​CAA​GAG​CGA​AGT​CC 3'
	R 5' CCC​TGC​TGT​GTC​TCG​AAA​AA 3'
Hgo	F 5' TTC​GAT​GAT​CGG​GAT​GTA​AAG 3'
	R 5' GGA​CAA​GTG​AGC​ACG​GTA​AAA 3'
CASK	F 5' GGA​CAT​CGG​TGC​GAA​TGA​GTA 3'
	R 5' GCT​GCC​GTC​GTA​ATC​TGC​TAT 3'
CG31674	F 5' GGT​GGT​CGG​GAC​ATA​CTC​AA 3'
	R 5' GTA​AGA​TCG​GCT​ACC​GCA​AC 3'
Lpin	F 5' TGGAGCGTCGCAACCTAA 3'
	R 5' GGCTTCTTCTCGCCCTGA 3'
BcDNA:RH44935	F 5' GTG​GCT​ACA​AGG​TGC​CTG​AAT 3'
	R 5' GTA​CTT​GGC​CAT​TTC​CAC​CTC 3'
Trp	F 5' AGG​GCA​CGG​ACA​AGT​TCA​A 3'
	R 5' TAT​GCT​CCA​GCA​GGA​TCA​CC 3'
Hppy	F 5' CAC​AGT​CAC​CAC​AAT​GCC​AAT​A 3'
	R 5' GGT​TCC​CGA​GCC​AAT​CTT​T 3'
Chchd2	F 5' TCA​TGG​ATT​GAC​CTC​GCT​GTT 3'
	R 5' TGG​AGA​TGG​TGG​CTC​TGC​TT 3'
Dmel\CG5510	F 5' CTGTTCGGCGACGGATTT 3'
	R 5' GCT​GAG​ATA​TGG​ATG​TTG​GTG​G 3'

### Western Blotting

The significant proteins were validated using western blotting. Male *D. melanogaster* from the control and RG group were euthanized at the age of 36 days. The test was repeated three times. Total protein was extracted from *D. melanogaster* with lysis buffer (Beyotime, Haimen, China) and Bullet Blender (NY, United States). Protein concentration was tested using the BCA protein assay reagent. GAPDH was used as the loading control. Samples were separated by 12% SDS-PAGE (Bio-Rad, Hercules, CA, United States) and transferred to polyvinylidene difluoride membranes (Millipore, United States). Membranes were blocked with 5% w/v nonfat dry milk (BD Biosciences, United States) and incubated with primary antibodies at 4°C overnight. The membranes were washed with Tris-buffered saline-Tween and incubated with horseradish peroxidase-labeled secondary antibodies (Proteintech, United States) for 1.5 h at 25°C. Finally, the enhanced chemiluminescence kit (GE Healthcare, United States) was used to visualize the immunobands. The protein bands were scanned using an imaging densitometer.

### Statistical Analyses

SAS software (version 9.2) was used for statistical analysis. Fisher’s LSD (least significant difference) was used for the analysis of life span and climbing ability test data. The data of qRT-PCR and Western blotting were analyzed using *t*-test. A *p* value of less than 0.05 was considered statistically significant.

## Result

### Red Ginseng on Prolongs the Life Span of Male *D. melanogaster*

**FIGURE 2:**
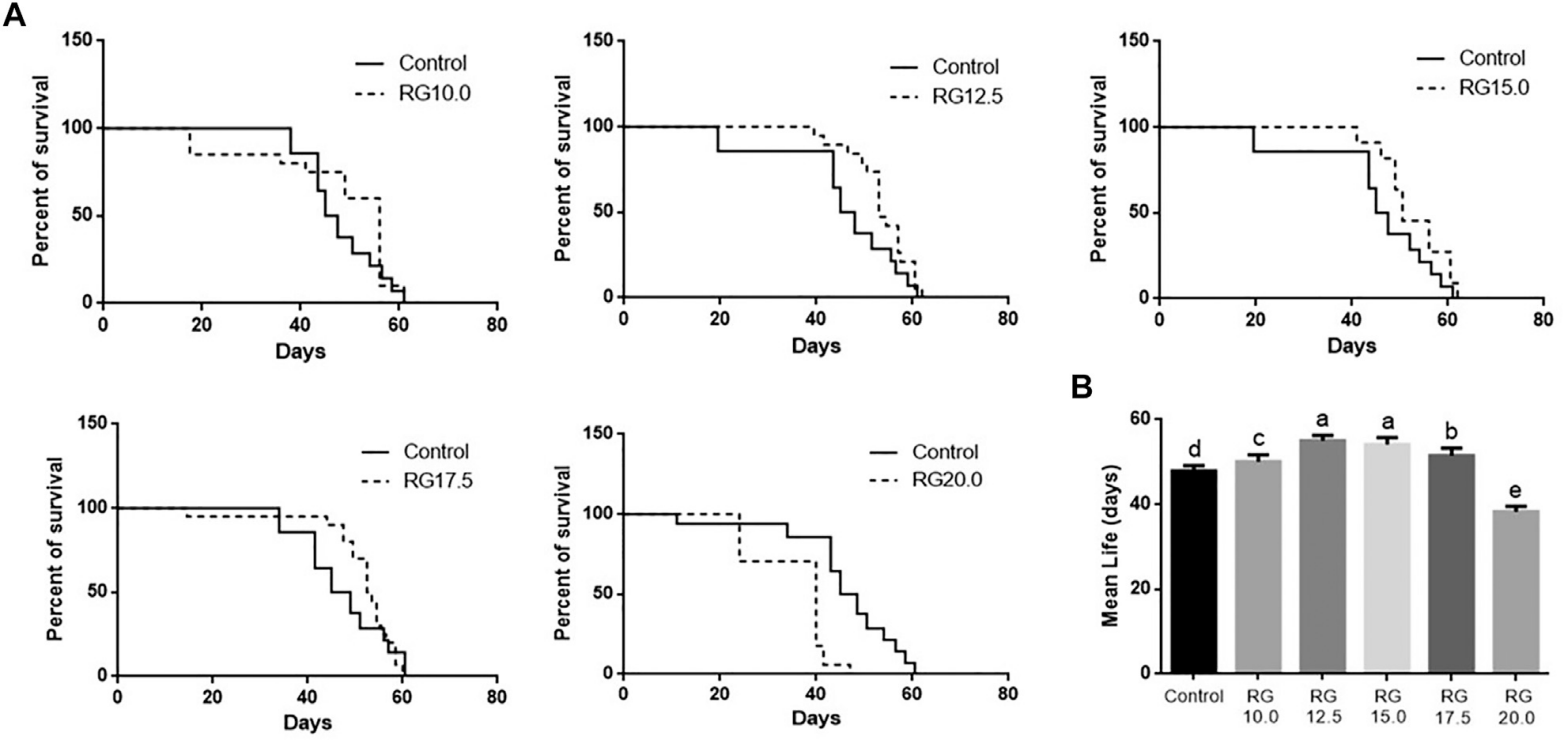
Red ginseng on prolonging life span of male *D. melanogaster*; B. Statistical analysis of data. The different lowercase letters mean significant difference at a 5% level with each treatment group.; Abbreviation: RG, red ginseng.

The male *D. melanogaster* in the RG group were fed RG at concentrations of 10.0, 12.5, 15.0, 17.5, or 20.0 mg/ml. The life spans of male *D. melanogaster* treated with 12.5 mg/ml RG and 15.0 mg/ml RG were significantly extended compared with the control group, while the life spans of male *D. melanogaster* treated with 20.0 mg/ml RG decreased (Figure 2). These results indicated that RG could prolong the life span of male *D. melanogaster* within the appropriate dose range, while an overdose of RG had a negative impact on the life span.

### Climbing Ability Test

**FIGURE 3:**
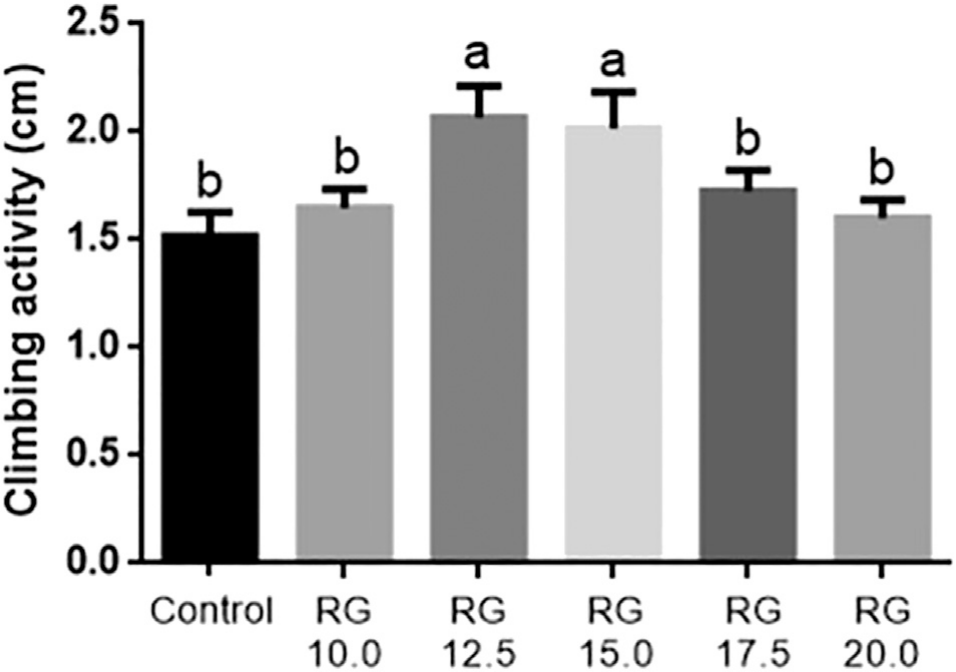
Climbing ability test.The different lowercase letters mean significant difference at a 5% level with each treatment group.; Abbreviation: RG, red ginseng.

The male *D. melanogaster* of the control and RG groups were tested at the age of 36 days. Climbing activity had already been successfully used to evaluate the rate of aging in *D. melanogaster.* The male *D. melanogaster* treated with 12.5 mg/ml and 15.0 mg/ml RG had better climbing activity compared with the control group (Figure 3). These results indicated that RG could slow down the rate of aging.

### Identification of Significantly Changed Proteins

**FIGURE 4:**
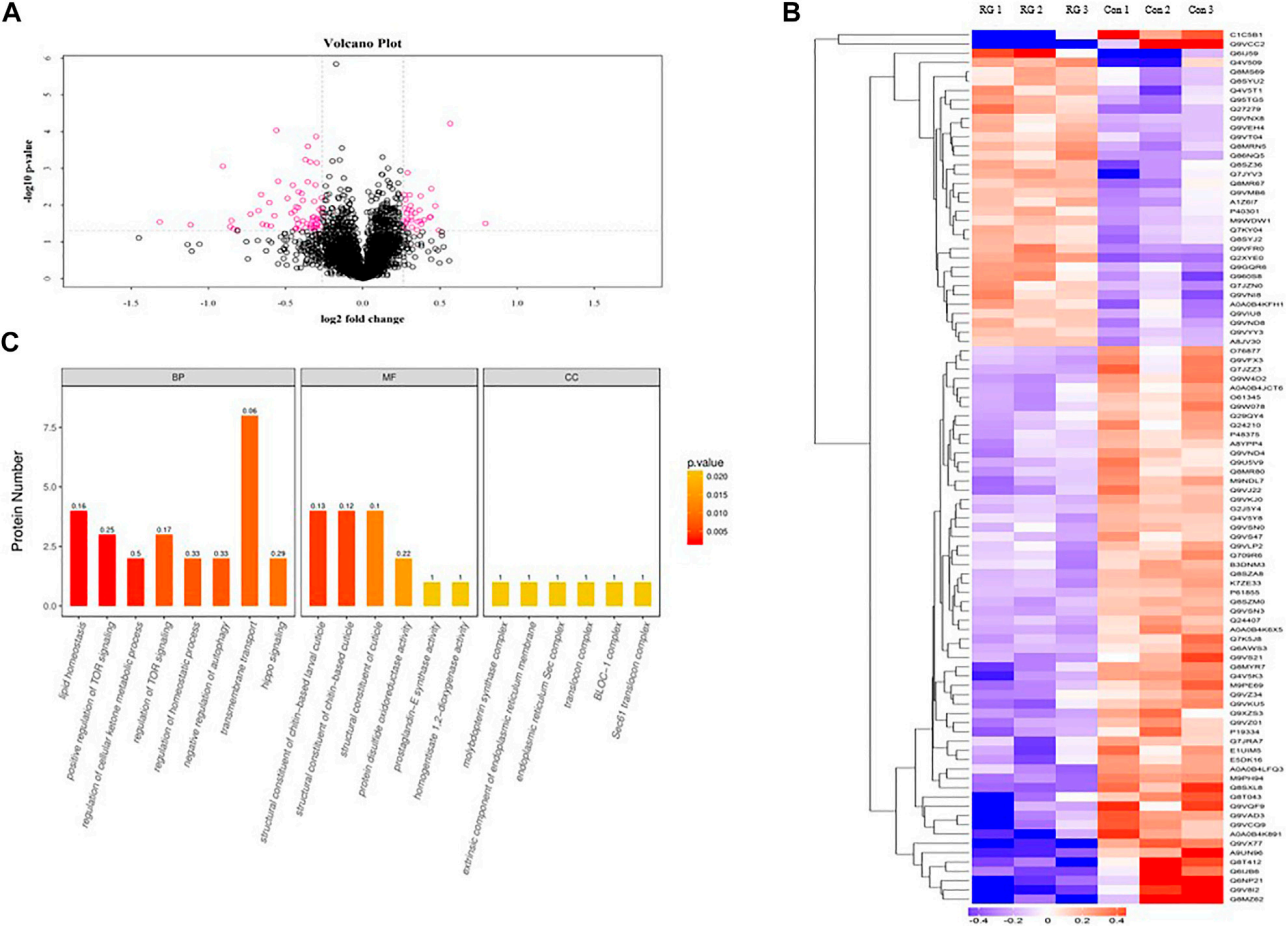
**(A)**. Volcano plot showed the significant changes in proteins expression; The pink dots were the differentially expressed proteins in the figure (the multiple change was greater than 1.2 times and *p* value <0.05). The black dots were the proteins which had not changed. **(B)**. Clustering heatmaps showed the significant changes in proteins expression; The red represented significantly up-regulated proteins. The blue represented significantly down-regulated proteins. Gray represented no protein quantitative information. **(C)**. The significantly changed proteins were analyzed by the Blast2GO program. The abscissa represented the enriched GO terms in the figure. Biological Process, BP; Molecular Function, MF; Cellular Component, CC; The ordinate represented the number of differentially expressed proteins. The color gradient represented the size of the *p* value. The closer the color was to the red, the smaller the *p* value, and the higher the significance level of the enrichment degree of the corresponding GO functional category. The label above the bar chart showed the enrichment factor (Enrichment factor represented the ratio of the number of differentially expressed proteins annotated to a GO function to the number of all identified proteins annotated to that GO function).; Abbreviation: RG, red ginseng; Con, control.

To investigate the effects of RG on male *D. melanogaster* life span, we treated male *D. melanogaster* with 12.5 mg/ml RG and used iTRAQ to identify protein changes. Using a threshold of >1.2 or <0.83 (*p* < 0.05), a total of 94 proteins were found to be differentially expressed between the RG and control groups. Of these, the expression of 32 proteins increased and the expression of 62 proteins decreased in the experimental group (Table 2). Clustering heatmaps and volcano plots showed significant changes in protein expression (Figures 4A,B).

**TABLE 2:** Identification of differential proteins in male *D. melanogaster*.

Entry	Protein ID	Gene name	RG/Control	*p*	Change
1	Q6IJ59	HDC15811	1.735026	0.031516984	Up
2	Q2XYE0	CG8343	1.480231	5.98782E-05	Up
3	Q4V509	CG10445	1.406139	0.049010132	Up
4	Q27279	Su(fu)	1.383591	0.010438172	Up
5	Q9VFR0	CG9286	1.359872	0.003587151	Up
6	Q7JYV3	CG12374	1.355848	0.020600274	Up
7	Q960S8	Unr	1.34977	0.021517929	Up
8	Q9VNI8	Hpr1	1.316567	0.025014078	Up
9	Q4V5T1	CG14661	1.300725	0.032815710	Up
10	Q8MRN5	CG5839	1.296939	0.005629237	Up
11	Q8SZ36	Vir-1	1.293305	0.026735257	Up
12	Q86NQ5	CG8773	1.289529	0.013560964	Up
13	Q8MR67	CG10472	1.278358	0.028262384	Up
14	A0A0B4KFH1	Muskelin	1.276112	0.046799449	Up
15	Q95TG5	Svil	1.264427	0.016375094	Up
16	Q9GQR6	Stai	1.245608	0.039443523	Up
17	Q9VND8	Rheb	1.245072	0.014024349	Up
18	Q7JZN0	Sec61beta	1.243214	0.025590847	Up
19	A1Z6I7	BubR1	1.242469	0.020906239	Up
20	Q9VEH4	Dmel\CG14325	1.238543	0.010173205	Up
21	A8JV30	Dmel\CG34327	1.233242	0.005286876	Up
22	Q9VMB6	Ent2	1.226665	0.01309915	Up
23	Q8SYJ2	ND-MLRQ	1.223182	0.025171248	Up
24	Q9VYY3	Uba5	1.222387	0.001308629	Up
25	Q9VIU8	Dmel\CG10132	1.217911	0.016758497	Up
26	Q7KY04	Rab4	1.212045	0.029160556	Up
27	Q8SYU2	RpL7-like	1.211114	0.031963084	Up
28	M9WDW1	Lds-RA	1.210729	0.007067795	Up
29	Q9VT04	Path	1.207497	0.012074239	Up
30	Q9VNX8	CG7414	1.205446	0.00520136	Up
31	P40301	Prosalpha2	1.201562	0.026267051	Up
32	Q8MS69	Dmel\CG9596	1.201365	0.043806106	Up
33	A8YPP4	CG30296	0.832942	0.015104015	Down
34	Q9VS47	Anon-WO0118547.349	0.83233	0.037428812	Down
35	Q4V5Y8	CG13309	0.83139	0.00628201	Down
36	Q9VSN0	Zasp66	0.829811	0.011645056	Down
37	Q29QY4		0.829644	0.023284602	Down
38	Q9VLP2	Dmel\CG7781	0.823622	0.023643643	Down
39	P48375	FK506-bp2	0.823203	0.013030252	Down
40	Q9VND4	Dmel\CG14671	0.813542	0.032412725	Down
41	P61855	Akh	0.813533	0.000702961	Down
42	B3DNM3		0.813445	0.023169705	Down
43	Q8MR80	EG:BACR42I17.2	0.811568	0.033391311	Down
44	A0A0B4JCT6	Kank	0.810175	0.039877023	Down
45	G2J5Y4	CG8446-RE	0.810138	0.000136072	Down
46	Q9VKJ0	Hgo	0.8088	0.002827784	Down
47	Q9U5V9	Su(*p*)	0.804879	0.040446674	Down
48	Q24407	ATPsynCf6	0.804015	0.007844585	Down
49	O61345	Peng	0.803825	0.020630303	Down
50	Q9VZ34	Dmel\CG2076	0.803381	0.048727204	Down
51	O76877	BcDNA:LD03613	0.803176	0.032438469	Down
52	Q24210	CASK	0.80112	0.041871672	Down
53	Q6AWS3	CG31674	0.798807	0.026233971	Down
54	Q709R6	Bocks	0.798735	0.02187602	Down
55	Q7JRA7	CG15096	0.793707	0.039789077	Down
56	Q9W078	Cpr62Bb	0.790797	0.033351008	Down
57	Q8SZM0	Cpr92F	0.789962	0.000668051	Down
58	K7ZE33	PUG	0.781302	0.002310275	Down
59	Q9VSN3	Cpr66D	0.781155	0.000250163	Down
60	Q7K5J8	Cpr57A	0.778778	0.031569703	Down
61	Q8SZA8	Fdx2	0.772951	0.000578374	Down
62	Q9VFX3	BcDNA:RE53127	0.772918	0.041415202	Down
63	E5DK16	Lpin	0.764213	0.026655127	Down
64	A0A0B4K6X5	Dmel\CG43093	0.763455	0.007419164	Down
65	Q9VZ01	BcDNA:RH44935	0.76059	0.004836408	Down
66	Q7JZZ3	CG13321	0.751225	0.046743486	Down
67	M9NDL7	Reps	0.749973	0.004348601	Down
68	P19334	trp	0.747794	0.03200816	Down
69	Q9W4D2	Rnp4F	0.747663	0.011693647	Down
70	Q9XZS3	CG13364	0.742911	0.023862935	Down
71	Q9VJ22	BEST:GH09876	0.741874	0.011097374	Down
72	E1UIM5	CG13675	0.741544	0.042732988	Down
73	Q9VS21	Dmel\CG15829	0.739049	0.044600699	Down
74	Q9VKU5	Dmel\CG6144	0.736171	0.014459095	Down
75	A0A0B4LFQ3	Hppy	0.730729	0.002866597	Down
76	M9PE69	Dmel\CG43740	0.725764	0.016526142	Down
77	Q8MYR7	PI4KIIIalpha	0.713236	0.006458911	Down
78	Q4V5K3	CG15653	0.683802	0.002240457	Down
79	M9PH94	Caz	0.678528	9.17871E-05	Down
80	Q9VCQ9	Dmel\CG6733	0.672357	0.019442633	Down
81	Q8T043	CG3409-RA	0.661867	0.036690684	Down
82	Q9VAD3	Vps13B	0.657683	0.008481404	Down
83	Q9VQF9	Snapin	0.649571	0.035362079	Down
84	Q6IJB6	HDC15303	0.637737	0.032958939	Down
85	Q8SXL8	Cln3	0.63377	0.005102953	Down
86	A0A0B4K891	CG8963	0.625873	0.014196561	Down
87	Q8T412	CG10749	0.60393	0.017466337	Down
88	A9UN96	CG18259	0.569743	0.047419921	Down
89	Q8MZ62	CG5103	0.559821	0.044463531	Down
90	Q9V8I2	CG5189	0.554082	0.025951457	Down
91	Q6NP21	CG12124	0.551103	0.038401808	Down
92	Q9VX77	Chchd2	0.533666	0.000874477	Down
93	C1C5B1	cact-RB	0.461301	0.034408467	Down
94	Q9VCC2	Dmel\CG5510	0.402157	0.028519357	Down

*p* value <0.05 was considered statistically significant.

### Bioinformatics Analysis of Significantly Changed Proteins

The significantly changed proteins were analyzed by the Blast2GO program and Fisher’s exact test (*p* < 0.05). The enriched GO terms were from the following three categories: cellular component, molecular function, and biological process. The biological process category was significantly enriched in GO for proteins involved in lipid homeostasis, positive regulation of TOR signaling, and regulation of cellular ketone metabolic process. The molecular function category was significantly enriched in GO for structural constituents of the chitin-based larval cuticle, protein disulfide oxidoreductase activity, and prostaglandin-E synthase activity. The cellular component category was significantly enriched in GO for the extrinsic component of the endoplasmic reticulum membrane, molybdopterin synthase complex, and translocation complex (Figure 4C). Significantly changed proteins were analyzed by KEGG. The identified pathways were protein processing in the endoplasmic reticulum, oxidative phosphorylation, and the mTOR signaling pathway.

### Validation of Significantly Changed Proteins by qRT-PCR

**FIGURE 5:**
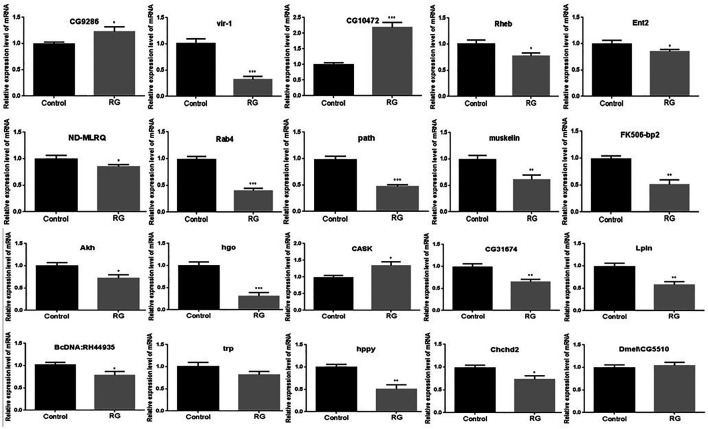
Validation of the significantly changed proteins by qRT-PCR. *p* value <0.05 was considered statistically significant.; Abbreviation: RG, red ginseng.

The mRNA levels of these differentially expressed proteins were tested by qRT-PCR. The mRNA level changes of CG9286, CG10472, FK506-bp2, Akh, hgo, CG31674, Lpin, BcDNA:RH44935, hppy, and Chchd2 were consistent with the changes analyzed by iTRAQ. No significant changes were evident in the levels of trp and Dmel\CG5510 between the control and RG groups (Figure 5).

### Validation of Proteins by Western Blotting

**FIGURE 6:**
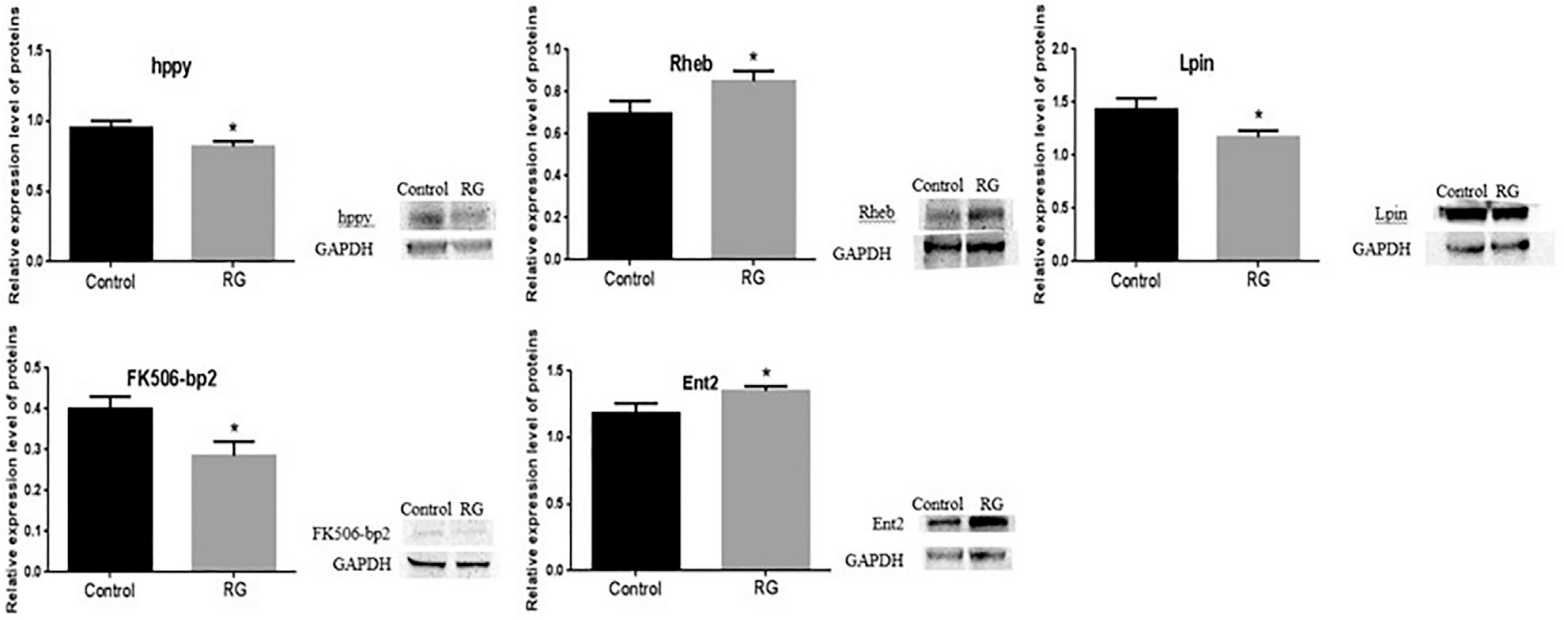
Expression of hppy, Rheb, Lpin, FK506-bp2 and Ent2 were confirmed using western blotting. *P* value < 0.05 was considered statistically significant.; Abbreviation: RG, red ginseng.

Expression of hppy, Lpin, Ent2, Rheb, and FK506-bp2 was tested by western blotting. The altered expressions of hppy, Lpin, Ent2, Rheb, and FK506-bp2 between the control and RG groups were consistent with the changes analyzed by iTRAQ. The expression of hppy, Lpin, and FK506-bp2 was downregulated and the expression of Ent2 and Rheb was upregulated (Figure 6).

### Effect of Hppy, Rheb, and Lpin on the PI3K/AKT/FoxO Pathway

**FIGURE 7:**
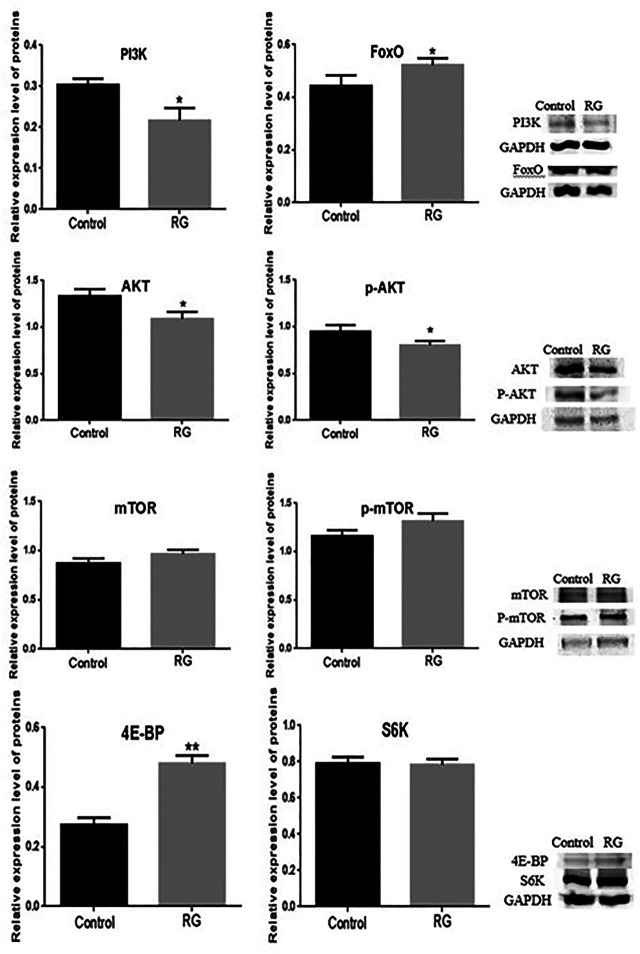
Expression of PI3K, AKT, p-AKT, mTOR, p-mTOR, 4E-BP, S6K and FoxO were confirmed using western blotting. *P* value < 0.05 was considered statistically significant.; Abbreviation: RG, red ginseng; Con, control; AKT, protein kinase B; mTOR, mechanistic target of rapamycin, PI3K, phosphatidylinositol 3 kinase; 4E-BP, eukaryotic translation initiation factor 4E binding protein; S6K, S6 protein kinase; FoxO, forkhead box O.

**FIGURE 8:**
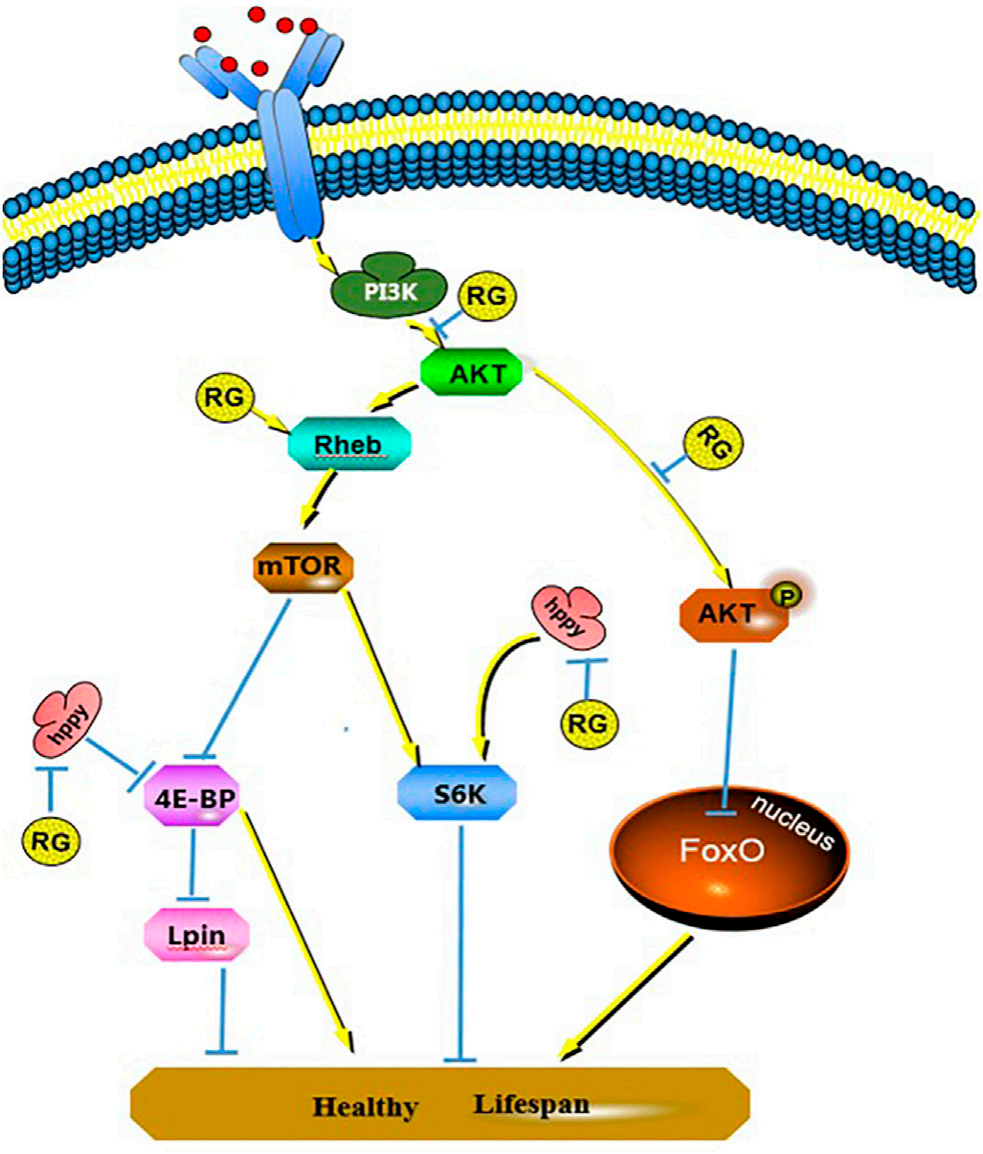
Changes of signal pathways induced by RG prolonging male *D. melanogaster* life span. *P* value < 0.05 was considered statistically significant.; Abbreviation: RG, red ginseng; Con, control; AKT, protein kinase B; mTOR, mechanistic target of rapamycin, PI3K, phosphatidylinositol 3 kinase; 4E-BP, eukaryotic translation initiation factor 4E binding protein; S6K, S6 protein kinase; FoxO, forkhead box O.

The differentially expressed proteins hppy, Rheb, and Lpin participated in the phosphatidylinositol 3 kinase (PI3K)/protein kinase B (AKT)/forkhead box O (FoxO) signaling pathway and regulated the life span of *D. melanogaster*. We tested the changes of PI3K, AKT, p-AKT, mTOR, p-mTOR, S6K, 4E-BP, and FoxO proteins using western blotting. The expression of PI3K, AKT, and p-AKT was decreased in the RG group (*p* < 0.05), and the expression of mTOR and p-mTOR did not change significantly. The expression of 4E-BP and FoxO increased significantly (*p* < 0.05) and S6K did not change (Figures 7, 8).

## Discussion

Red ginseng prolongs the life span of *D. melanogaster*, but the mechanism has not been elucidated. In this study, iTRAQ was used to identify protein changes in senile (36 days old) male *D. melanogaster*. The iTRAQ examination revealed that a total of 94 proteins were differentially expressed in the RG group. Of these, the expression of 32 proteins increased and the expression of 62 proteins decreased. qRT-PCR revealed variations in the mRNA levels of CG9286, CG10472, FK506-bp2, Akh, hgo, CG31674, Lpin, BcDNA: RH44935, hppy, and Chchd2, which were consistent with the iTRAQ results. No significant changes were seen in the levels of trp and Dmel\CG5510 between the control and RG groups. The mRNA expression of vir-1, Rheb, Ent2, ND-MLRQ, Rab4, path, muskelin, and CASK after qRT-PCR detection were not consistent with iTRAQ results (Figure 4). This inconsistency in changes may be because mRNA is regulated by many regulatory factors during translation. The altered expression of hppy, Lpin, Ent2, Rheb, and FK506-bp2 found in western blotting were consistent with the results of iTRAQ.

The differentially expressed protein hppy, which is homologous to human MAP4K3, regulates various signaling pathways, including the mTOR pathway (Lam et al., 2010). The mTOR pathway plays an important role in the process of *Drosophila* senescence. A decrease in MAP4K3 level can reduce the activity of mTOR (Bryk et al., 2010). S6 protein kinase (S6K) and translation inhibitor (4E-BP) are targets of mTOR activity, and MAP4K3 also regulates the activity of S6K and 4E-BP (Weichhart 2018). The effect of 4E-BP on extending life span has been shown in *Drosophila* before (Hay 2011). In our study, western blotting results showed that the expression of hppy in male *D. melanogaster* was decreased after RG treatment. The expression of mTOR, p-mTOR, and S6K did not change. The expression of 4E-BP increased significantly (*p* < 0.05). The findings indicated that the decreased expression of hppy upregulated the expression of 4E-BP, directly inhibited protein synthesis, and participated in the regulation of life extension. In addition, MAP4K3 can also activate the JNK pathway and induce cell apoptosis. Based on these results, the decreased expression of hppy may inhibit the JNK pathway (Lam et al., 2010).

The differentially expressed protein Rheb, a homolog of Ras GTPase, participates in the PI3K/AKT/mTOR signaling pathway and upregulates Rheb activated mTOR activity (Karassek et al., 2010). Inhibiting mTOR activity and reducing protein synthesis can prolong the life span of organisms, including yeast, *D. melanogaster*, nematodes, and mice (Alic and Partridge 2011). However, long-term inhibition of mTOR activity can lead to inhibition of wound healing, anemia, proteinuria, pneumonia, and hypercholesterolemia (Lamming et al., 2012). The balance of the mTOR pathway is essential for cell health. In this study, the results showed that the expression of Rheb in male *D. melanogaster* was increased after RG treatment, and the expression of PI3K, AKT, and p-AKT was decreased in the RG group (*p* < 0.05). The expression of p-mTOR increased slightly in the RG group, but the difference was not significant (*p* > 0.05). Inactivation of AKT directly activates the FoxO family of proteins, which reduces oxidative stress, repairs DNA damage, and inhibits premature aging and cellular senescence (Zhang et al., 2011). Therefore, we tested the changes of FoxO protein, and the results showed that the expression of FoxO was increased (*p* < 0.05). The findings indicated that RG prolonged the life span of male *D. melanogaster* by regulating the PI3K/AKT/FoxO pathway. The mTOR pathway remained relatively balanced and was not overactivated. In addition, Rheb can reduce reactive oxygen species (ROS) and oxidative damage independently of the mTOR pathway (Ashraf et al., 2019). On feeding *D. melanogaster* RG, Rheb may reduce the oxidative damage and prolong the life span.

The differentially expressed protein Lpin, a homolog of human lipin, is a lipid protein regulated by the mTOR pathway. Aging is accompanied by the accumulation of Lpin (Romic et al., 2017). The expression of Lpin can be decreased by inhibiting mTOR and upregulating the expression of 4E-BP (Guo et al., 2019; Reue and Wang, 2019). The iTRAQ, qRT-PCR, and western blotting results showed that the expression of Lpin was decreased and 4E-BP was increased. The decreased expression of Lpin indicated that there was no excessive accumulation of lipids in the senile flies.

The differentially expressed protein Fk506-bp2 changed significantly, which is a binding protein. Fk506-bp2 is sensitive to oxidative stress and easily decomposes with the ryanodine receptor (Kreko-Pierce et al., 2016). The results showed that the expression of FK506-bp2 decreased, which may reduce the sensitivity to oxidative stress and oxidative damage.

The differentially expressed protein CG31674 is an oxidoreductase involved in oxidative stress (Yi et al., 2007; Fernandez-Ayala et al., 2010). Our results showed that the expression of CG31674 decreased. It was speculated that the activity of the redox reaction and oxidative damage may have decreased. Oxidative damage can induce aging. Significant prolongation of a healthy life span requires a reduction of all aging processes of an organism (Avril, et al., 1984).

Red ginseng prolonged the life span of male *D. melanogaster* through a complex biological process and may be used as a potential anti-aging drug. The iTRAQ examination revealed that a total of 94 proteins were differentially expressed in the RG group. Many proteins do not have primary antibodies and western blotting confirmatory experiments were not executed. In addition, *D. melanogaster* is a model organism and non-mammal. We will continue to study RG’s effect on prolonging life span and PI3K/AKT/FoxO pathway in mammals.

## Conclusion

In this study, we explored the pathways that RG may participate in when extending the life span of *D. melanogaster*, and the results showed that the PI3K/AKT/FoxO pathway was involved. In addition, 4E-BP expression increased and participated in the regulation of life span.

## Data Availability

Raw mass spectrometry data were submitted to China National Genomics Data Center (CNCB-NGDC, https://bigd.big.ac.cn/omix/) under BioProject accession number PRJCA004725.

## References

[B1] Alic N. Partridge L. (2011). Death and Dessert: Nutrient Signalling Pathways and Ageing. Curr. Opin. Cel Biol. 23, 738–743. 10.1016/j.ceb.2011.07.006 PMC4335171 21835601

[B2] Allocca M. Zola S. Bellosta P. (2018). The Fruit Fly, Drosophila melanogaster: Modeling of Human Diseases (Part II), Drosophila melanogaster - model for recent advances in genetics and therapeutics [M]. London: *InTech Open*, 131–156.

[B3] Ashraf S. Kim B. J. Park S. Park H. Lee S.-H. (2019). RHEB Gene Therapy Maintains the Chondrogenic Characteristics and Protects Cartilage Tissue from Degenerative Damage during Experimental Murine Osteoarthritis. Osteoarthritis and cartilage 27, 1508–1517. 10.1016/j.joca.2019.05.024 31229684

[B31] Avril D. W. Anthony D. B. Alexander H. (1984). Molecular biology of aging [M]. New York, Plenum Press, 16–17.

[B4] Bryk B. Hahn K. Cohen S. M. Teleman A. A. (2010). MAP4K3 Regulates Body Size and Metabolism in Drosophila. Develop. Biol. 344, 150–157. 10.1016/j.ydbio.2010.04.027 20457147

[B5] Fernandez-Ayala D. J. Chen S. Kemppainen E. O'Dell K. M. C. Jacobs H. T. (2010). Gene Expression in a Drosophila Model of Mitochondrial Disease. PLoS One 5, e8549. 10.1371/journal.pone.0008549 20066047 PMC2798955

[B6] Guo Z. Cheng X. Feng X. Zhao K. Zhang M. Yao R. (2019). The mTORC1/4EBP1/PPARγ Axis Mediates Insulin-Induced Lipogenesis by Regulating Lipogenic Gene Expression in Bovine Mammary Epithelial Cells. J. Agric. Food Chem. 67, 6007–6018. 10.1021/acs.jafc.9b01411 31060359

[B7] Ham S. W. Kim J.-K. Jeon H.-Y. Kim E.-J. Jin X. Eun K. (2019). Korean Red Ginseng Extract Inhibits Glioblastoma Propagation by Blocking the Wnt Signaling Pathway. J. Ethnopharmacology 236, 393–400. 10.1016/j.jep.2019.03.031 30878548

[B8] Hay N. (2011). Interplay between FOXO, TOR, and Akt. Biochim. Biophys. Acta (Bba) - Mol. Cel Res. 1813, 1965–1970. 10.1016/j.bbamcr.2011.03.013 PMC3427795 21440577

[B9] He Y. Jasper H. (2014). Studying Aging in Drosophila. Methods 68, 129–133. 10.1016/j.ymeth.2014.04.008 24751824 PMC4066732

[B10] Hou W. Pei J. Wang Y. P. Zhang J. Zheng H. S. Cui R. (2020). Anti-ageing Effects of Red Ginseng on Female *Drosophila melanogaster* . J. Cel Mol Med. 24, 3751. 10.1111/jcmm.15029 PMC7131930 32022406

[B11] Karassek S. Berghaus C. Schwarten M. Goemans C. G. Ohse N. Kock G. (2010). Ras Homolog Enriched in Brain (Rheb) Enhances Apoptotic Signaling*. J. Biol. Chem. 285, 33979–33991. 10.1074/jbc.m109.095968 20685651 PMC2962498

[B12] Kim M. S. (2013). Korean Red Ginseng Tonic Extends Lifespan in *D. melanogaster* . Biomolecules Ther. 21, 241–245. 10.4062/biomolther.2013.024 PMC3830124 24265871

[B13] Kreko-Pierce T. Azpurua J. Mahoney R. E. Eaton B. A. (2016). Extension of Health Span and Life Span in Drosophila by S107 Requires the Calstabin Homologue FK506-BP2. J. Biol. Chem. 291, 26045–26055. 10.1074/jbc.m116.758839 27803160 PMC5207075

[B14] Lam D. Shah S. de Castro I. P. Loh S. H. Y. Martins L. M. (2010). Drosophila Happyhour Modulates JNK-dependent Apoptosis. Cell Death Dis 1, e66. 10.1038/cddis.2010.44 21364671 PMC3032524

[B15] Lamming D. W. Ye L. Katajisto P. Goncalves M. D. Saitoh M. Stevens D. M. (2012). Rapamycin-induced Insulin Resistance Is Mediated by mTORC2 Loss and Uncoupled from Longevity. Science 335, 1638–1643. 10.1126/science.1215135 22461615 PMC3324089

[B16] Liu Q.-X. Zhang W. Wang J. Hou W. Wang Y.-P. (2018). A Proteomic Approach Reveals the Differential Protein Expression in *Drosophila melanogaster* Treated with Red Ginseng Extract (Panax Ginseng). J. Ginseng Res. 42, 343–351. 10.1016/j.jgr.2017.04.006 29983616 PMC6026366

[B17] Liu X. Wang J. Gao L. Liu H. Liu C. (2017). iTRAQ-Based Proteomic Analysis of Neonatal Kidney from Offspring of Protein Restricted Rats Reveals Abnormalities in Intraflagellar Transport Proteins. Cell Physiol Biochem 44, 185–199. 10.1159/000484626 29130966

[B19] Minois N. Khazaeli A. A. J W. (2001). Locomotor Activity as a Function of Age and Life Span in *Drosophila melanogaster* Overexpressing Hsp70. Exp. Gerontol. Curtsinger 36 (7), 1137–1153. 10.1016/s0531-5565(00)00263-1 11404055

[B20] Ramesh T. Kim S.-W. Sung J.-H. Hwang S.-Y. Sohn S.-H. Yoo S.-K. (2012). Effect of Fermented Panax Ginseng Extract (GINST) on Oxidative Stress and Antioxidant Activities in Major Organs of Aged Rats. Exp. Gerontol. 47, 77–84. 10.1016/j.exger.2011.10.007 22075532

[B21] Reue K. Wang H. (2019). Mammalian Lipin Phosphatidic Acid Phosphatases in Lipid Synthesis and beyond: Metabolic and Inflammatory Disorders. J. Lipid Res. 60, 728–733. 10.1194/jlr.s091769 30804008 PMC6446709

[B22] Romic S. Krskova K. Olszanecki R. Balazova L. Lory V. Koricanac G. (2017). Obesity- and Age-Related Alterations in FAT/CD36 Translocation and Lipin-1 Subcellular Localization in Skeletal Muscle of the Zucker Rats. Gen. Physiol. Biophys. 36, 399–406. 10.4149/gpb_2017010 28653652

[B23] Weichhart T. (2018). mTOR as Regulator of Lifespan, Aging, and Cellular Senescence: A Mini-Review. Gerontology 64, 127–134. 10.1159/000484629 29190625 PMC6089343

[B24] Wisniewski J. R. Zougman A. Nagaraj N. Mann M. (2009). Universal Sample Preparation Method for Proteome Analysis. Nat. Methods 6, 359–362. 10.1038/nmeth.1322 19377485

[B25] Wu X. Pei J. Wang Y. Zhang J. Zheng H. Cui R. (2017). Ginseng: An Nonnegligible Natural Remedy for Healthy Aging. Aging Dis. 8 (6) 708, 10.14336/AD.2017.0707 29344412 PMC5758347

[B26] Yi C. H. Sogah D. K. Boyce M. Degterev A. Christofferson D. E. Yuan J. (2007). A Genome-wide RNAi Screen Reveals Multiple Regulators of Caspase Activation. J. Cel Biol 179, 619–626. 10.1083/jcb.200708090 PMC2080898 17998402

[B27] Yu H. Wang X. Xu J. Ma Y. Zhang S. Yu D. (2017). iTRAQ-Based Quantitative Proteomics Analysis of Molecular Mechanisms Associated with *Bombyx mori* (Lepidoptera) Larval Midgut Response to BmNPV in Susceptible and Near-Isogenic Strains. J. Proteomics 165, 35–50. 10.1016/j.jprot.2017.06.007 28624519

[B28] Yuan S. M. (2015). Potential Cardioprotective Effects of Ginseng Preparations. Pak J. Pharm. Sci. 28, 963–968 . 26004730

[B29] Zhang H. Xu S. Piao C. Zhao X. Tian Y. Cui D. (2018). Post-planting Performance, Yield, and Ginsenoside Content of Panax Ginseng in Relation to Initial Seedling Size. Ind. Crops Prod. 125, 24–32. 10.1016/j.indcrop.2018.08.091

[B30] Zhang X. Tang N. Hadden T. J. Rishi A. K. (2011). Akt, FoxO and Regulation of Apoptosis. Biochim. Biophys. Acta (Bba) - Mol. Cel Res. 1813, 1978–1986. 10.1016/j.bbamcr.2011.03.010 21440011

